# The association of egg consumption with blood pressure levels and glycated hemoglobin in Spanish adults according to body mass index

**DOI:** 10.1038/s41598-022-21772-6

**Published:** 2022-10-19

**Authors:** Arthur Eumann Mesas, Miriam Garrido-Miguel, Rubén Fernández-Rodríguez, Sofía Fernández-Franco, Cristina Lugones-Sánchez, Luis García-Ortiz, Vicente Martínez-Vizcaíno

**Affiliations:** 1 Health and Social Research Center, Universidad de Castilla-La Mancha, 16071 Cuenca, Spain; 2 Universidade Estadual de Londrina, Londrina, Paraná 86057-970 Brazil; 3 Faculty of Nursing, Universidad de Castilla-La Mancha, C/ Campus Universitario , s/n (Edificio Benjamín Palencia), 02006 Albacete, Spain; 4 Grupo Avícola Rujamar, San Lorenzo de la Parrilla, 16770 Cuenca, Spain; 5 Unidad de Investigación en Atención Primaria de Salamanca (APISAL), Gerencia de Atención Primaria de Salamanca, Gerencia Regional de Salud de Castilla y León (SACyL), Instituto de Investigación Biomédica de Salamanca (IBSAL), 37005 Salamanca, Spain; 6 Departamento de Ciencias Biomédicas y del Diagnóstico, Universidad de Salamanca, 37007 Salamanca, Spain; 7 RedIAPP: Red Española de Investigación Para Actividades Preventivas y Promoción de la Salud en Atención Primaria, Barcelona, Spain; 8 Facultad de Ciencias de la Salud, Universidad Autónoma de Chile, 1101 Talca, Chile

**Keywords:** Biomarkers, Cardiology, Endocrinology, Risk factors

## Abstract

The objective of this study was to evaluate the association of egg consumption with blood pressure (BP) and glycated hemoglobin (HbA1c). In addition, it was assessed whether this association changes according to body weight status. This cross-sectional study is based on multicenter data from Spanish adult participants in the EVIDENT II trial. Egg consumption was assessed with a Food Frequency Questionnaire, and data on BP and HbA1c were collected using standardized procedures. Linear regression and ANCOVA models adjusted for the main confounders were performed. The analyses were stratified by body weight status. A total of 668 participants were analyzed (mean age 52.4 ± 11.8 years, 62.3% women). Compared with lower consumption, higher egg consumption was associated with lower systolic (ß =  − 6.15 ± 1.74; *p*-for-trend = 0.017), diastolic (ß =  − 4.41 ± 1.03; *p*-for-trend = 0.002), and mean arterial pressure (ß =  − 4.99 ± 1.17; *p*-for-trend = 0.003) and with lower HbA1c (ß =  − 0.19 ± 0.06; *p*-for-trend = 0.019) levels. These associations lost statistical significance in the adjusted analyses. The results did not vary by body weight status. In conclusion, consumption of up to 1 egg per day is not associated with BP or HbA1c, even in overweight or obese individuals. Our findings suggested that this frequency of egg consumption is safe as part of a healthy diet and lifestyle for cardiometabolic risk.

## Introduction

Eggs are considered one of the most profitable and sustainable foods due to their high nutritional value and the presence of bioactive nutrients^1^. However, the controversy about whether and how egg consumption is associated with cardiovascular disease (CVD) risk has persisted, so that accumulating evidence does not yet allow firm recommendations on egg consumption to be made^2^. Eggs also contribute considerable amounts of dietary cholesterol, which could lead to a negative impact on cardiometabolic risk markers^3^. Conversely, although higher egg consumption has been associated with increased CVD and general mortality^3^, a recent umbrella review concluded that increased egg consumption is not associated with CVD risk in the general population^4^. Moreover, recent meta-analyses have not confirmed this harmful association in prospective studies^5,6^, and one stated that up to one egg per day could be added as part of a healthy diet without worrying about increasing the risk of CVD^7^. A lower coronary artery disease incidence or mortality was associated with egg consumption^8^. Apart from not negatively influencing CVD, consuming more than 1 egg/d has been associated with a significant reduction in the risk of coronary artery disease^9^. We recently reported that there is no association between egg consumption and lipid profile even in individuals with obesity, diabetes, hypertension or ≥ 1 chronic metabolic diseases^10^. In addition to the lipid profile, cardiometabolic risk is also closely related to blood pressure and glycemic control due to their individual and combined implications for endothelial function and inflammation levels.

Similar to what is observed with lipids, it is still unclear whether egg consumption increases, decreases or even is not associated with blood pressure and glycemic control. Recent studies reported that higher egg consumption increases the risk of hypertension^11^ and type 2 diabetes^12^. However, in meta-analyses of randomized clinical trials, it was observed that the levels of blood pressure^13^ and the risk for developing type 2 diabetes^14^ did not increase in the group consuming more eggs compared with those consuming fewer eggs. Last, a meta-analysis of observational studies reported that egg consumption was associated with a lower risk of hypertension in the overall multivariable-adjusted analysis^15^.

Therefore, the primary purpose of the present study was to analyze the relationship between egg consumption and blood pressure parameters (i.e., systolic blood pressure [SBP], diastolic blood pressure [DBP] and mean arterial pressure [MAP]) and glycated hemoglobin (HbA1c) in a sample of Spanish adults. Moreover, with the additional aim of controlling and exploring in depth the role of body mass index (BMI) in these associations, we employed two methodological approaches: 1) considering that body weight is strongly associated with both dietary intake and cardiometabolic risk, we calculated egg consumption according to intake in grams per day per kg of body weight (g/day/kg of BW)^16^, dividing the intake into quartiles of consumption; and 2) with the aim of providing a potential public health recommendation/message about egg intake effects on blood pressure and glycemic control according to body weight status, we conducted subgroup analyses by body weight status.

## Results

A total of 668 individuals were analyzed (mean age 52.4 ± 11.8 years, 62.3% women). The mean ± standard deviation of egg consumption was 0.30 ± 0.16 g/day/kg of BW, equivalent to 2.5 eggs of 60 g consumed weekly by an adult of 70 kg of BW. As presented in Table 1, considering the same reference (i.e., weekly egg intake of 60 g for a 70 kg of BW adult), the distribution of participants according to quartiles of consumption resulted in a 1st quartile consuming none or less than 1.2 eggs per week, while the 4th quartile, corresponding to high consumption, comprised from > 3.2 up to 6.9 eggs per week. None of the participants included in the present analyses consumed more than 7 eggs per week.

**Table 1:** Characteristics of the study participants by the number of eggs consumed per week per kg of body weight.

Characteristic	Total	Egg consumption (g/day/kg of body weight)				*p* value^a^
		1st quartile (0 to 0.14)	2nd quartile (> 0.14 to 0.32)	3rd quartile (> 0.32 to 0.39)	4th quartile (> 0.39 to 0.84)	
Number of 60 g eggs/week for an individual with 70 kg of body weight		0 to 1.2	> 1.2 to 2.6	> 2.6 to 3.2	> 3.2 to 6.9	
Total, n (%)	668 (100.0)	167 (25.0)	178 (26.6)	158 (23.7)	165 (24.7)	
Age (years)	52.4 ± 11.8	51.9 ± 12.5^a^	51.9 ± 11.6^a^	56.1 ± 9.1^b^	50.1 ± 12.7^a^	< 0.001
Female, n (%)	416 (62.3)	97 (58.1)	79 (44.4)	106 (67.1)	134 (81.2)	< 0.001
University studies, n (%)	127 (19.0)	36 (21.6)	34 (19.1)	28 (17.7)	29 (17.6)	0.871
Body mass index (kg/m^2^)	27.9 ± 4.8	28.4 ± 4.4^a^	30.5 ± 5.6^b^	27.5 ± 2.8^a^	24.9 ± 4.2^c^	< 0.001
Current smoker, n (%)	131 (19.6)	29 (22.1)	41 (31.3)	26 (19.8)	35 (26.7)	0.373
Alcohol drinker, n (%)	523 (78.3)	126 (24.1)	145 (27.7)	123 (23.5)	129 (24.7)	0.601
High adherence to the Mediterranean Diet, n (%)^b^	214 (32.0)	44 (26.3)	54 (30.3)	57 (36.1)	59 (35.8)	0.175
Moderate-to-vigorous physical activity (min/week)	461.0 ± 215.1	454.6 ± 201.3	450.9 ± 221.4	461.6 ± 223.4	477.8 ± 214.6	0.672
Total energy intake (kcal/day)	2471.4 ± 776.5	2205.6 ± 851.3^a^	2502.5 ± 723.8^b^	2512.7 ± 645.8^b^	2667.4 ± 800.6^b^	< 0.001
Hypertension, n (%)^c^	224 (33.5)	54 (24.1)	81 (36.2)	56 (25.0)	33 (14.7)	< 0.001
Systolic blood pressure (mmHg)	123.9 ± 16.4	123.7 ± 14.6^a^	128.9 ± 18.1^b^	125.5 ± 15.1^a^	117.5 ± 15.3^c^	< 0.001
Diastolic blood pressure (mmHg)	75.8 ± 9.8	75.8 ± 8.9^a^	79.2 ± 10.4^b^	76.5 ± 8.7^a^	71.4 ± 9.5^c^	< 0.001
Mean arterial pressure (mmHg)	91.9 ± 11.1	91.8 ± 9.9^a^	95.7 ± 11.9^b^	92.9 ± 9.9^a^	86.8 ± 10.7^c^	< 0.001
Type 2 diabetes, n (%)^d^	49 (7.3)	16 (32.7)	14 (28.6)	9 (18.4)	10 (20.4)	0.507
Glycated hemoglobin (%)	5.50 ± 0.56	5.56 ± 0.74^a^	5.51 ± 0.51^a^	5.54 ± 0.43^a^	5.38 ± 0.49^b^	0.014

Values are means ± standard deviations, except when indicated “n (%)”.

^a^Chi-squared test for categorical variables and ANOVA for continuous variables. Different letters indicate significant differences between quartiles of egg consumption identified with the Bonferroni post hoc test. ^b^Scores higher than 9 indicate high adherence to the Mediterranean Diet. ^c^Systolic blood pressure ≥ 140 and/or diastolic blood pressure ≥ 90 mmHg or using antihypertensive drugs). ^d^HbA1c ≥ 6.5% or using antidiabetic drugs.

Compared to lower consumption (1st quartile), higher consumption (4th quartile) was characterized by a higher frequency of women (58.1 vs. 81.2%, respectively), lower BMI (28.4 vs. 24.9 kg/m^2^, respectively), higher total energy intake (2205.6 vs. 2667.4 kcal/day, respectively) and lower frequency of hypertension (24.1 vs. 14.7%, respectively). Regarding blood pressure parameters, individuals classified as higher egg consumers presented lower SBP, DBP and MAP than those who consumed fewer eggs per kg of BW (*p* < 0.001). These same blood pressure parameters were higher in the 2nd quartile than in the 1st, 3rd and 4th quartiles. Finally, HbA1c levels were significantly lower (*p* = 0.014) in participants in the 4th quartile (5.38%) than in those in the 1st quartile (5.56%) of egg consumption (Table 1).

The correlation analyses presented in Table 2 showed that egg consumption was inversely associated with SBP (*r* =  − 0.092), DBP (*r* =  − 0.120), MAP (*r* =  − 0.116) and HbA1c (*r* =  − 0.090).

**Table 2:** Bivariate correlation between egg consumption and blood pressure parameters and glycated hemoglobin.

Variables	Egg consumption	Systolic blood pressure	Diastolic blood pressure	Mean arterial pressure	Glycated hemoglobin
Egg consumption	1.00				
Systolic blood pressure	− 0.092*	1.00			
Diastolic blood pressure	− 0.120**	0.717**	1.00		
Mean arterial pressure	− 0.116**	0.912**	0.940**	1.00	
Glycated hemoglobin	− 0.090*	0.165**	0.112**	0.147**	1.00

Values indicate the correlation coefficient (r).

*p < 0.050, **p < 0.010. Egg consumption is in grams/day/kg of body weight unit, systolic, diastolic blood pressure and mean arterial pressure are in mmHg, and glycated hemoglobin is in percentage.

Unadjusted linear regression (Model 1) comparing higher versus lower consumption (i.e., 4th vs. 1st quartiles) showed that the higher the consumption of eggs, the lower the SBP (ß =  − 6.15 ± 1.74; *p*-for-trend = 0. 017) and DBP (ß =  − 4.41 ± 1.03; *p*-for-trend = 0.002), MAP (ß =  − 4.99 ± 1.17; *p*-for-trend = 0.003) and HbA1c (ß =  − 0.19 ± 0.06; *p*-for-trend = 0.019) (Table 3). The associations between egg consumption and DBP and mean arterial pressure remained after adjusting for age, sex and educational level (Model 2) but lost statistical significance when adjusted for BMI, total energy intake and adherence to the Mediterranean Diet (Model 3). Additionally, the results of the unadjusted logistic regression (Model 1) presented in Table 4 showed that, compared to the 1st quartile of egg consumption, there was an increased likelihood of hypertension in those in the 2nd quartile, while those in the 4th quartile were less likely to have hypertension. These associations lost statistical significance in Model 3, adjusted for sociodemographic and diet-related covariates. No association between egg consumption and type 2 diabetes was observed in the unadjusted or adjusted models (Table 4).

**Table 3:** Linear regression models of the association of egg consumption with blood pressure parameters and glycated hemoglobin.

Outcomes	1st quartile	2nd quartile	3rd quartile	4th quartile	*p*-for-trend
*Model 1* (*unadjusted*)					
Systolic blood pressure	Reference	5.17 ± 1.70**	1.79 ± 1.76	− 6.15 ± 1.74**	0.017
Diastolic blood pressure	Reference	3.37 ± 1.01**	0.72 ± 1.04	− 4.41 ± 1.03**	0.002
Mean arterial pressure	Reference	3.97 ± 1.15**	1.08 ± 1.18	− 4.99 ± 1.17**	0.003
Glycated hemoglobin	Reference	− 0.06 ± 0.06	− 0.03 ± 0.06	− 0.19 ± 0.06**	0.019
*Model 2*					
Systolic blood pressure	Reference	4.08 ± 1.48*	0.91 ± 1.54	− 2.79 ± 1.52	0.238
Diastolic blood pressure	Reference	2.83 ± 0.96*	0.43 ± 1.00	− 3.00 ± 0.99*	0.025
Mean arterial pressure	Reference	3.25 ± 1.03*	0.59 ± 1.07	− 2.93 ± 1.06*	0.051
Glycated hemoglobin	Reference	− 0.06 ± 0.06	− 0.10 ± 0.06	− 0.16 ± 0.06	0.737
*Model 3*					
Systolic blood pressure	Reference	2.61 ± 1.47	1.81 ± 1.51	− 1.14 ± 1.58	0.729
Diastolic blood pressure	Reference	1.71 ± 0.95	0.97 ± 0.98	− 1.26 ± 1.02	0.484
Mean arterial pressure	Reference	2.01 ± 1.02*	1.25 ± 1.04	− 0.88 ± 1.09	0.787
Glycated hemoglobin	Reference	− 0.11 ± 0.06	− 0.06 ± 0.06	− 0.07 ± 0.06	0.238
*Model 4*					
Systolic blood pressure	Reference	2.66 ± 1.47	1.92 ± 1.51	0.06 ± 1.57	0.674
Diastolic blood pressure	Reference	1.72 ± 0.95	1.02 ± 0.98	− 1.17 ± 1.02	0.717
Mean arterial pressure	Reference	2.03 ± 1.01*	1.32 ± 1.04	− 0.76 ± 1.08	0.632
Glycated hemoglobin	Reference	− 0.12 ± 0.06	− 0.06 ± 0.06	− 0.06 ± 0.06	0.643

Values indicate the coefficient ± standard error obtained through linear regression models. Model 1: unadjusted. Model 2: adjusted for age (continuous, years), sex (male, female) and education level (primary or secondary studies, university studies). Model 3: Model 2 adjusted for body mass index (continuous, Kg/m^2^), total energy intake (continuous, kcal/day) and adherence to the Mediterranean Diet (continuous, MEDAS score). Model 4: Model 3 adjusted for smoking status (nonsmoker or former smoker, current smoker), alcohol intake (nondrinker, current alcohol drinker) and moderate-to-vigorous physical activity (continuous, minutes/week).

*p < 0.050, **p < 0.001.

**Table 4:** Logistic regression models of the association between egg consumption and hypertension (yes or no) and type 2 diabetes (yes or no).

Outcomes	1st quartile	2nd quartile	3rd quartile	4th quartile	*p*-for-trend
*Model 1* (*unadjusted*)					
Hypertension	Reference	1.75 (1.13, 2.71)*	1.15 (0.73, 1.82)	0.52 (0.32, 0.86)*	0.107
Type 2 diabetes	Reference	0.81 (0.38, 1.71)	0.57 (0.24, 1.33)	0.61 (0.27, 1.38)	0.564
*Model 2*					
Hypertension	Reference	1.99 (1.19, 3.12)*	0.97 (0.58, 1.63)	0.66 (0.37, 1.17)	0.336
Type 2 diabetes	Reference	0.70 (0.32, 1.52)	0.52 (0.22, 1.23)	0.84 (0.35, 1.97)	0.815
*Model 3*					
Hypertension	Reference	1.61 (0.93, 2.79)	1.32 (0.76, 2.31)	1.29 (0.67, 2.45)	0.267
Type 2 diabetes	Reference	0.54 (0.24, 1.23)	0.81 (0.32, 2.04)	1.61 (0.61, 4.22)	0.339
*Model 4*					
Hypertension	Reference	1.63 (0.93, 2.85)	1.39 (0.79, 2.43)	1.33 (0.69, 2.55)	0.201
Type 2 diabetes	Reference	0.60 (0.26, 1.38)	0.80 (0.31, 2.04)	1.68 (0.62, 4.53)	0.410

Values indicate the odds ratio (95% confidence interval) obtained through logistic regression models. Hypertension was defined as systolic blood pressure ≥ 140 and/or diastolic blood pressure ≥ 90 mmHg or using antihypertensive drugs. Type 2 diabetes was defined as HbA1c ≥ 6.5% or using antidiabetic drugs. Model 1: unadjusted. Model 2: adjusted for age (continuous, years), sex (male, female) and education level (primary or secondary studies, university studies). Model 3: Model 2 adjusted for body mass index (continuous, Kg/m^2^), total energy intake (continuous, kcal/day) and adherence to the Mediterranean Diet (continuous, MEDAS score). Model 4: Model 3 adjusted for smoking status (nonsmoker or former smoker, current smoker), alcohol intake (nondrinker, current alcohol drinker) and moderate-to-vigorous physical activity (continuous, minutes/week). *p < 0.05.

**Figure 1:**
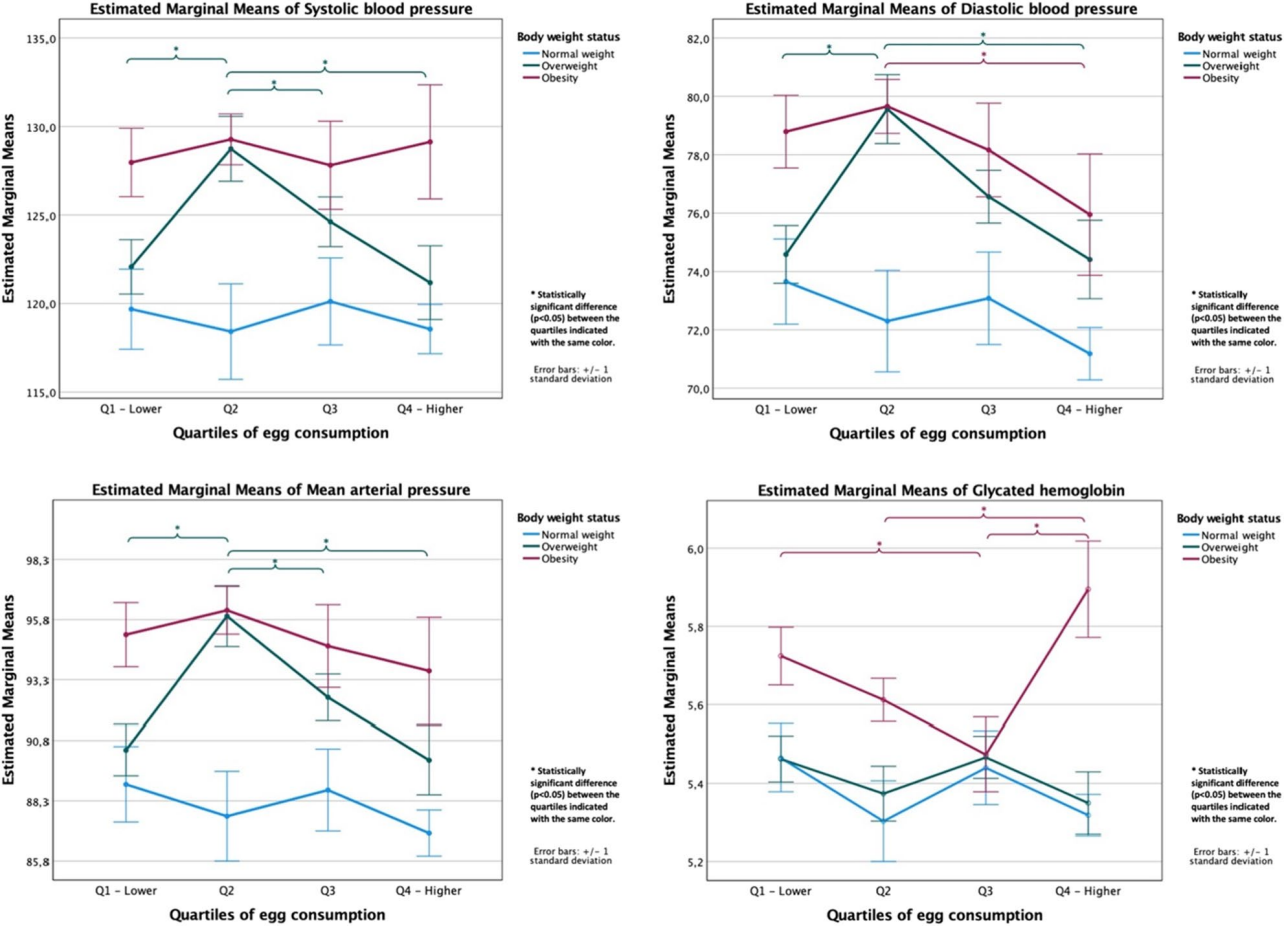
Estimated marginal means (± 1 standard deviation) of blood pressure and glycated hemoglobin according to body weight status obtained with ANCOVA models adjusted for age (continuous, years), sex (male, female), BMI (continuous, Kg/m^2^), education level (primary or secondary studies, university studies), smoking status (nonsmoker or former smoker, current smoker), alcohol intake (nondrinker, current alcohol drinker), total energy intake (continuous, kcal/day), adherence to the Mediterranean Diet (continuous, MEDAS score) and moderate-to-vigorous physical activity (continuous, minutes/week).

The absence of a significant association when comparing the 1st and 4th quartiles of egg consumption was also observed in the analyses stratified by body weight status (Fig. 1). Although blood pressure and HbA1c parameter levels were generally higher in obese individuals than in those of normal weight, the estimated marginal means of these indicators did not differ significantly when comparing obese individuals in the 1st or in the 4th quartiles of egg consumption.

## Discussion

In this study, in Spanish adults, egg consumption was negatively associated with SBP, DBP, MAP, and HbA1c levels, although these associations lost statistical significance when considering the confounding effect of sociodemographic and diet-related aspects such as BMI, total energy intake and adherence to the MD. Furthermore, no differences were found between normal weight, overweight and obese individuals. Taken together, our findings corroborate the evidence that these individual characteristics exert a relevant role in addition to those from the bioactive components of the egg. Viewed another way, they support that within a balanced and good quality dietary pattern, the consumption of up to 1 egg per day is safe because it does not imply negative effects on blood pressure and blood sugar control even in overweight or obese individuals.

Our results regarding eggs and blood pressure are overall consistent with those from other cross-sectional and prospective studies carried out with different adult populations in several countries^17–20^. However, other studies found contradictory results both in the US^12,21^ and in other countries^11,22^. The Westernized dietary pattern, which is predominant among US adults and is increasing in other developed countries^23^, has been pointed to as one of the main reasons for the discrepancy between studies conducted in different countries^21,24,25^. On the one hand, compared with other dietary patterns, the Westernized diet is characterized by a higher intake of total daily energy and foods associated with hypertension, such as red meat, sausages and ultra-processed foods rich in saturated fatty acids and sodium^26^. For eggs, the average number of servings per week is very different from country to country^6^. In that sense, part of the population of Spain, like the participants in the present study, adhere to the Mediterranean Diet, a dietary pattern consistently associated with lower cardiovascular risk^27^. It is possible that the increase in blood pressure observed in the US studies and other countries is accounted for by the other unhealthy foods consumed in the usual diet by the average higher egg consumption in the US. Therefore, dietary aspects such as unhealthy dietary foods and accompaniments, mode of cooking, and body composition may play a more important role in the possible risk of hypertension than egg consumption in isolation. This more comprehensive view of the dietary effects on health must consider the diet as a whole, but isolated foods have been strengthened in recent years^28^.

Regarding the possible relationship between egg consumption and glycemic control, our results indicate that, similar to what we observed with peripheral blood pressure parameters, the frequency of egg consumption appears to be unrelated to blood sugar levels. Importantly, our results are based on the percentage of HbA1c, a more suitable biochemical indicator of glycemic control than fasting blood glucose because it is less subject to fluctuations due to recent dietary intake and fasting time^29^. Consistent with our findings, the available evidence from prospective studies^25,30,31^ and a randomized clinical trial^28^ predominantly supports that consuming eggs does not interfere with glycemic control. Furthermore, as we have seen in unadjusted analyses, other authors found associations compatible with possible benefits of egg consumption on blood sugar levels. For example, in a cross-sectional study of more than 3 thousand Chinese adults, a dose–response curve showed that with the increase in egg consumption, the risk of type 2 diabetes first increased and then decreased^32^. Some authors argue that the protective effect of the eggs consumed on glycemia could be due to the higher intake of specific bioactive components of this food involved in the pathophysiology of type 2 diabetes^33^, specifically choline and polyunsaturated fatty acids (linolenic and docosahexaenoic acids)^34^. While this biological pathway is quite reasonable and certainly requires in-depth research, our data support that the results of these studies could be confounded by variables included in our analyses and not considered by those authors, such as BMI and behavioral variables such as total energy intake, quality of diet and the amount of physical activity.

The possible interactions found in other studies with respect to sex^5,19^ were not corroborated by our findings since we did not detect effect modification between men and women for the associations of egg consumption with blood pressure or HbA1c. The same was observed with BMI in our analyses, which is contrary to some studies that reported different findings for people with and without obesity, with reference to egg consumption both in relation to blood pressure^19^ and diabetes risk^35^. It is worth mentioning that in our analyses, specifically in the obese group, the levels of blood pressure and HbA1c were significantly higher in the 2nd quartile than in the 1st quartile, decreasing again in the 3rd and 4th quartiles. We can conjecture that this occurred because, compared to the 1st quartile, the 2nd quartile shows a higher proportion of men (41.9 vs. 55.6%, respectively), higher mean BMI (28.4 vs. 30.5 kg/m^2^, respectively) and higher total daily energy intake (2,205.6 vs. 2,502.5 kcal/day, respectively). However, these unexpected results remain not completely justified since our analyses were adjusted for these variables. Future studies are needed to better understand why egg consumption might present a nonlinear relationship with peripheral blood pressure levels^20^, particularly in obese individuals, as observed in the present analyses.

Some limitations must be considered for the correct interpretation of the present results. First, the cross-sectional design prevents us from drawing conclusions about the temporal relationship between consuming eggs and variations in blood pressure levels and HbA1c. Therefore, longitudinal studies with subgroups of normal weight, overweight, and obese participants exposed to different amounts of egg consumption are needed to confirm that consuming up to 1 daily egg is safe for blood sugar and blood pressure control even in the presence of excess weight. Second, although this is a multicenter study with a sample obtained from health centers in different regions of Spain, it cannot be inferred that the results apply to all adults in the country. Moreover, the participants in the present study were free of advanced CVD, cancer, or other major physical or mental disorders. Therefore, caution must be taken in extrapolating the results to populations with sociodemographic and behavioral characteristics, as well as health conditions different from those analyzed. Third, if we examined objective parameters of body weight and height, blood pressure and glycated hemoglobin with high validity and reliability, information on diet and other covariates was obtained subjectively. Although the applied questionnaires collected data subject to recall and information bias, this is a limitation shared with most epidemiological studies with considerable sample sizes, such as ours. Finally, although our analyses included adjustment for several variables, residual confounding remains a potential limitation (such as comorbidities and the use of drugs for treatment, family history of hypertension and diabetes mellitus, specific consumption of other foods with an effect on blood pressure [refined salt, ultra-processed foods, etc.] and on the glycemic level [sweets, pasta, soft drinks, etc.], among others).

The results of this cross-sectional study with Spanish adults allow us to conclude that egg consumption is not associated with blood pressure and glycemic control when considering sociodemographic, lifestyle and BMI as confounding factors. No associations were confirmed even in overweight or obese individuals. These findings are consistent with previous research that found a significant association between higher egg consumption and potential benefits for human health. In addition, our study might reinforce the moderate consumption of eggs along with a balanced healthy diet and lifestyle.

## Methods

### Study design and participants

This was a cross-sectional analysis of data from baseline measurements of the EVIDENT II trial (NCT02016014), a multicenter, randomized double-blind clinical trial that aimed to develop and validate a smartphone application and to evaluate the effect of adding this tool to a standardized intervention designed to improve adherence to the Mediterranean diet and to physical activity^36^. The study included six groups of the Research Network on Preventive Activities and Health Promotion (REDIAPP) in Bilbao, Cuenca, Zaragoza, Valladolid, Barcelona, and Salamanca (Spain). This trial included adult men and women (aged 18 to 70 years) free of advanced cardiovascular disease, cancer, and other major physical or mental disorders. Face-to-face and individual interviews, as well as anthropometric measurements, were performed in a research center by previously trained investigators. Recruitment, data collection and measurement procedures have been described elsewhere^36^. The study was approved by the Ethics Committee of Salamanca University Hospital (Spain), and all participants gave written informed consent according to the general recommendations of the Declaration of Helsinki.

Of the 833 participants who were initially examined, 127 were excluded because of a lack of data on HbA1c, 30 were excluded because of a lack of dietary data, and 8 were excluded because of missing data on any of the covariates that were considered. Thus, the present analyses were based on a subsample of 668 individuals (80.2%) in which all dataset variables were measured. The characteristics of the participants included in the present analysis were overall very similar to those from the total initial population (Table S1, Supplementary material).

### Study variables

#### Exposure

Egg consumption was obtained with a 137-item Food Frequency Questionnaire (FFQ-137) that has been validated in a population of elderly people at high cardiovascular risk in Spain^37^. An incremental scale with 6 levels, from "1 to 3 times/month" to "2 to 3 times/day", was used to collect information on food consumption frequencies. To convert participants' responses on egg consumption frequency to consumption in g/day, it was considered i) that 1 standard egg weighs 60 g and ii) that 1 egg was consumed according to the midpoint of the frequency of each category. For example, individuals who reported consuming eggs 1 to 3 times/month were considered to consume 2 (the midpoint between 1 and 3) 60 g/month eggs, which is equivalent to 4 g/day (2 × 60 g/30 days = 4 g/day). In addition, because body weight (BW) is an important variable to consider when studying diet-related cardiometabolic risk factors, we used the unit of measurement in g/day/kg of BW, the same currently used by the European Food Safety Authority (EFSA), to recommend dietary guidelines^16,38^.

#### Outcomes

Three measurements of systolic (SBP) and diastolic (DBP) blood pressure were performed with a validated Omron M10-IT model sphygmomanometer (Omron Healthcare, Kyoto, Japan), and the average of the last two measurements was considered for each participant. Mean arterial pressure (MAP) was calculated as DBP + (0.333 x [SBP-DBP]). The measurements were made on the participant’s dominant arm in a seated position after at least 5 min of rest with a cuff of appropriate size, as determined by measurement of the upper arm circumference and following the recommendations of the European Society of Hypertension^39^.

To evaluate HbA1c, blood samples were obtained from the cubital vein between 8.00 and 9.00 a.m. after the individuals had fasted and abstained from smoking, alcohol, and caffeinated beverages for the previous 12 h. Blood samples were collected at the respective health centers, and all samples were analyzed at the city hospital that participates in the external quality assurance programs of the Spanish Society of Clinical Chemistry and Molecular Pathology.

#### Covariates

Information was also collected on potentially confounding covariates of the association between egg consumption and the outcomes, including sociodemographic variables (age [continuous, years], sex [male, female], educational level [none, primary or secondary studies, university studies]), total energy intake (continuous, in Kcal/day, obtained with the FFQ-137 questionnaire), adherence to the Mediterranean diet (continuous, obtained with the validated 14-point Mediterranean Diet Adherence Screener [MEDAS score]^40^), tobacco consumption (nonsmoker or former smoker, current smoker), alcohol intake (nondrinker, current alcohol drinker), and moderate-to-vigorous leisure-time physical activity (continuous, in minutes/week, measured with the International Physical Activity Questionnaire –IPAQ). BMI (continuous, kg/m^2^) was obtained with objective measures of body weight divided by height squared. According to the cutoffs for BMI defined by the World Health Organization, individuals were classified as normal weight (< 25 kg/m^2^), overweight (≥ 25 to < 30 kg/m^2^) or obese (≥ 30 kg/m^2^).

### Statistical analysis

To differentiate the lowest and the highest egg consumption, quartiles of consumption were established in the statistical analyses. The lowest intake category (from 0 to 0.14 g of egg/day/kg of BW) was used as the reference category. To give a more applicable sense of the unit of measurement of egg consumption, if an individual with a BW of 70 kg and a standard egg weight of 60 g is considered, the 1st quartile varies from 0 eggs to approximately 1 egg consumed per week (0.14 g/day/kg of BW = 0.14 × 70 kg × 7 days/60 g = 1.2 or ≈ 1 egg/week).

Statistical analysis included a description of the study population and the variables analyzed in total and by quartiles of egg consumption. First, the normal distribution of continuous variables was examined using both statistical (Kolmogorov–Smirnov test) and graphical (normal probability plots) methods. Then, chi-square tests were used for categorical variables, and ANOVA was used for continuous variables to compare the mean differences of each outcome variable according to the categories of egg consumption. Pairwise multiple comparisons were examined using the post hoc Bonferroni test. Then, the Pearson correlation test was applied considering the continuous variables of egg intake (g/d/kg of BW), SBP, DBP and MAP (mmHg) and HbA1c (%).

Linear regression models were used to analyze the association between egg consumption in quartiles of consumption (independent variable) and each of the blood pressure parameters (SBP, DBP and MAP) and HbA1c (dependent variables). Initially, an unadjusted model (Model 1) was performed separately for each dependent variable. In sequence, this model was adjusted for age (continuous, years), sex (male, female) and education level (primary or secondary studies, university studies) (Model 2). The next model was adjusted for all covariates of Model 2 adjusted for body mass index (continuous, Kg/m^2^), total energy intake (continuous, kcal/day) and adherence to the Mediterranean Diet (continuous, MEDAS score) (Model 3). Finally, the last model included all covariates of Model 3 and, in addition, was adjusted for smoking status (nonsmoker or former smoker, current smoker), alcohol intake (nondrinker, current alcohol drinker) and moderate-to-vigorous physical activity (continuous, minutes/week) (Model 4). To examine whether there was a linear trend in the association between egg consumption and the dependent variables, the aforementioned unadjusted and adjusted models were repeated after replacing the categorical variable in quartiles with the continuous variable of egg consumption in g/day/kg of BW.

Logistic regression models were also performed considering the dependent variables hypertension (SBP ≥ 140 and/or DBP ≥ 90 mmHg or using antihypertensive drugs) or type 2 diabetes (HbA1c ≥ 6.5% or using antidiabetic drugs). Similar to the procedure used in linear models, we repeated the unadjusted and adjusted models using the same set of potential confounders.

As other studies reported effect modification in the association between egg consumption and cardiometabolic risk by sex^5^ and body weight status^19^, we tested whether there was a first-order interaction with these variables. For each of them, we used the 2-log likelihood test, which compares the models without and with the corresponding interaction term. No interaction was found between egg consumption and sex and BMI considering all the dependent variables analyzed (*p* for interaction > 0.10 for all variables tested) (data not shown).

Finally, we performed analysis of covariance (ANCOVA) models for each dependent variable considering the quartiles of egg consumption and body weight status as fixed factors. These models included age, sex, education level, smoking status, alcohol intake, total energy intake, adherence to the Mediterranean Diet and moderate-to-vigorous physical activity as covariates. Although no interaction was observed between egg consumption and BMI, to make the results more transparent and to reinforce the main message of the study, estimated marginal means (± 1 standard deviation) of blood pressure parameters and HbA1c are represented in Fig. 1 according to the body weight status of the participants.

All analyses were carried out with the IBM SPSS program (version 28), and a *p* value < 0.05 was considered statistically significant.

## Supplementary Information

Supplementary Information.

## Data Availability

The data that support the findings of this study are available from the corresponding author (M.G.-M.) upon reasonable request.
