# Supplementary material for: The association of egg consumption with blood pressure levels and glycated hemoglobin in Spanish adults according to body mass index
Source: Sci Rep. 2022 Oct 19;12:17465. doi: 10.1038/s41598-022-21772-6 (PMC9582218; doi:10.1038/s41598-022-21772-6)
Supplement: Supplementary file 1 — Supplementary Information. [file 41598_2022_21772_MOESM1_ESM.pdf]

SUPPLEMENTARY MATERIAL

Table S1. Characteristics of the participants included in the initial population and in the sample analyzed in the present study.

Characteristic	Total initial population	Sample analyzed in the present study
Total, n (%)	883 (100.0)	668 (80.2)
Age (years)	51.9 ± 12.0	52.4 ± 11.8
Female, n (%)	517 (62.1)	416 (62.3)
University studies, n (%)	249 (29.9)	127 (19.0)
Body mass index (kg/m^2)	27.8 ± 4.9	27.9 ± 4.8
Current smoker, n (%)	172 (20.7)	131 (19.6)
Alcohol drinker, n (%)	637 (78.4)	523 (78.3)
High adherence to the Mediterranean Diet, n (%)[a]	231 (31.3)	214 (32.0)
Moderate-to-vigorous physical activity (min/week)	452.3 ± 217.5	461.0 ± 215.1
Total energy intake (kcal/day)	2473.7 ± 785.9	2471.4 ± 776.5
Hypertension, n (%)[b]	278 (33.4)	224 (33.5)
Systolic blood pressure (mmHg)	123.9 ± 16.1	123.9 ± 16.4
Diastolic blood pressure (mmHg)	76.2 ± 9.9	75.8 ± 9.8
Mean arterial pressure (mmHg)	92.1 ± 11.1	91.9 ± 11.1
Type 2 diabetes, n (%)[c]	62 (7.4)	49 (7.3)
Glycated hemoglobin (%)	5.50 ± 0.56	5.50 ± 0.56

Values are means ± standard deviations, except when indicated "n (%)". [a] Scores higher than 9 indicate high adherence to the Mediterranean Diet. [b] Systolic blood pressure ≥140 and/or diastolic blood pressure ≥90 mmHg or using antihypertensive drugs). [c] HbA1c ≥6.5% or using antidiabetic drugs.

The **EVIDENT 3 Investigators Group** comprised the following:

Unidad de Investigación de Atención Primaria de Salamanca (APISAL): Luis García-Ortiz (PI), José I Recio-Rodríguez, Cristina Lugones-Sánchez, Manuel A. Gómez-Marcos, Emiliano Rodríguez-Sánchez, Olaya Tamayo-Morales, Rosario Alonso-Domínguez, Natalia Sánchez-Aguadero, Susana González-Sánchez, Ángela de Cabo-Laso, Carmela Rodríguez-Martín, Carmen Castaño-Sánchez, Benigna Sánchez-Salgado, Jesús González-Sánchez, María C. Patino-Alonso, José A. Maderuelo-Fernández, Leticia Gómez-Sánchez, and Inés Llamas-Ramos.

Centro de Salud Torreramona de Zaragoza (Health Service of Aragón): Natividad González-Viejo, José Félix Magdalena-Belio, Luis Otegui-Ilarduya, Francisco J. Rubio-Galán, Cristina I. Sauras-Yera, Amor Melguizo-Bejar, María J. Gil-Train, Marta Iribarne-Ferrer, Olga Magdalena-González, Miguel A. Lafuente-Ripollés, M Mar Martínez, and Pilar Jiménez-Marcén.

Centro de Salud Cuenca I (Health Service of Castilla-La Mancha): Fernando Salcedo-Aguilar, Fructuoso Muelas-Herraiz, María A. Molina-Morate, Amparo Pérez-Parra, Fernando Madero, Ángel García-Imbroda, José M. Izquierdo, and María L. Monterde.

Universidad de Castilla-La Mancha (University of Castilla-La Mancha): Vicente Martínez-Vizcaíno, Alba Soriano-Cano, Diana Patricia Pozuelo-Carrascosa, Esther Gálvez-Adalia, Alicia del Saz-Lara, and Ana Díez-Fernandez.

Centro de Salud Sta. Ponça de Palma de Mallorca (Health Service of Balear Islands): José I. Ramírez-Manent, José L. Ferrer-Perelló, José E. Romero-Palmer, Manuel Sarmiento-Cruz, Guillermo Artigues, Jitka Mudrychova, María Albaladejo-Blanco, Margarita I. Moyá-Seguí, Cristina Vidal-Ribas, Patricia Lorente-Montalvo, Isabel Torrens-Darder, María M. Torrens-Darder, and Lucía Pascual Calleja.

Centro de Salud San Pablo de Valladolid (Health Service of Castilla y León): María J. Álvarez-Miguel, María D. de Arriba-Gómez, María A Rodríguez-Fernández, Isabel Arranz-Hernando, Silvia Ramos-de la Torre, Amparo Arqueaga-Luengo, María E. Moreno-Moreno, Agustina Marcos-García, Nora Manrique-Vinagre, Nieves Palomo-Blázquez, José L. Montalvillo-Montalvillo, María E. Fernández-Rodríguez, Alejandro González-Moro, Marta Santiago-Pastor, María I Pérez-Concejo, and Aurora Rubio-Fernández.

Centro de Salud Casa del Barco de Valladolid (Health Service of Castilla y León): Amparo Gómez-Arranz, Carmen Fernández-Alonso, Daniel Rodríguez-Domínguez, Irene Repiso-Gento, Aventina de la Cal-de la Fuente, Rosa Aragón-García, Miguel A. Díez-Garcia, Elisa Ibañes-Jalón, Ines Castrillo-Sanz, Ana M. Corcho-Castaño, Esther Jiménez-López, Daniel Correa-González, Lucía Barruso-Villafaina, Isabel Peña-García, Dolores Escudero-Terrón, Pilar Mena-Martín, Rosario Fraile-Gómez, Alberto Alonso-Gómez, Pilar Urueña, Francisca Martínez-Bermejo, Concepción Hernández-San José, Manuela Nuñez-Gómez, Patricia Sanz-Capdepont, Ana I Pazos-Revuelta, Sofía Pérez-Niño, and María E. Junquera-del Pozo.

CGB Computer Company, Salamanca, Spain, contributed to the technical development of the EVIDENT 3 application.
